# Effectiveness of Integrated Diabetes Care Interventions Involving Diabetes Specialists Working in Primary and Community Care Settings: A Systematic Review and Meta-Analysis

**DOI:** 10.5334/ijic.6025

**Published:** 2022-05-12

**Authors:** Reetu Zarora, Jincy Immanuel, Tawanda Chivese, Freya MacMillan, David Simmons

**Affiliations:** 1 School of Medicine, Western Sydney University, Diabetes Obesity and Metabolism Translational Research Unit, Macarthur Clinical School, Campbelltown, New South Wales, AU; 2 Department of Population Medicine, College of medicine, QU Health Qatar University, Doha, QA; 3 School of Health Science, Diabetes Obesity and Metabolism Translational Research Unit, The Translational Health Research Institute, Western Sydney University, Campbelltown, New South Wales, AU; 4 School of Medicine, Western Sydney University, Diabetes Obesity and Metabolism Translational Research Unit, The Translational Health Research Institute, Macarthur Clinical School, Campbelltown, New South Wales, AU

**Keywords:** integrated health care systems, multidisciplinary care, primary health care, diabetes mellitus, cost-effectiveness, clinical outcome

## Abstract

**Introduction:**

Evidence that integrated diabetes care interventions can substantially improve clinical outcomes is mixed. However, previous systematic reviews have not focussed on clinical effectiveness where the endocrinologist was actively involved in guiding diabetes management.

**Methods:**

We searched EMBASE, COCHRANE, MEDLINE, SCOPUS, CINAHL, Google Scholar databases and grey literature published in English language up to 25 January 2021. Reviewed articles included Randomised Controlled Trials (RCTs) and pre-post studies testing the effectiveness on clinical outcomes after ≥6 months intervention in non-pregnant adults (age ≥ 18 years) with type 1 or type 2 diabetes mellitus. Two reviewers independently extracted data and completed a risk of bias assessment. Appropriate meta-analyses for each outcome from RCTs and pre-post studies were performed. Heterogeneity was assessed using the *I^2^* statistic and Cochran’s Q and publication bias assessed using Doi plots. Studies were not pooled to estimate the cost-effectiveness as the cost outcomes were not comparable across trials/studies.

**Results:**

We reviewed 4 RCTs and 12 pre-post studies. The integrated care model of diabetes specialists working with primary care health professionals had a positive impact on HbA1c in both RCTs and pre-post studies and on systolic blood pressure, diastolic blood pressure, total cholesterol and weight in pre-post studies. In the RCTs, interventions reduced HbA1c (–0.10% [–0.15 to –0.05]) (–1.1 mmol/mol [–1.6 to –0.5]), versus control. Pre-post studies demonstrated improvements in HbA1c (–0.77% [–1.12 to –0.42]) (–8.4 mmol/mol [–12.2 to –4.6]), systolic blood pressure (–3.30 mmHg [–5.16 to –1.44]), diastolic blood pressure (–3.61 mmHg [–4.82 to –2.39]), total cholesterol (–0.33 mmol/L [–0.52 to –0.14]) and weight (–2.53 kg [–3.86 to –1.19]). In a pre-post study with no control group only 4% patients experienced hypoglycaemia after one year of intervention compared to baseline.

**Conclusions:**

Integrated interventions with an active endocrinologist involvement can result in modest improvements in HbA1c, blood pressure and weight management. Although the improvements per clinical outcome are modest, there is possible net improvements at a holistic level.

## Introduction

Diabetes is a global health priority affecting 463 million adults globally, with a further 374 million people at increased risk of developing the condition [1]. The prevalence of diabetes is projected to increase globally to 700 million by 2045 [1]. Diabetes takes a significant toll on health budgets with globally 760 billion USD expended on diabetes in 2019, and this expected to grow to a projected 845 billion USD by 2045 [1,2]. Diabetes can cause chronic complications and is associated with poor health outcomes, higher healthcare costs, and premature mortality [1].

Diabetes is a chronic and complex disease and early interventions, and adequate treatment can delay/prevent the onset of complications. Integrated care brings together organisations providing different levels of care and can be beneficial in people with diabetes who have multiple comorbidities to provide them with consistent management by a multidisciplinary team over a sustained period [3]. Lack of integrated care can make the care process disorganised [4]. Integrated care for diabetes can be defined as an inter-professional coordination, delivering patient centred care according to their multidimensional needs and improving patient experience by shared decision-making [5]. An article published in 1982 and a position statement by the American Diabetes Association in 1998 recommended that people with diabetes should receive treatment by a multidisciplinary team [6,7]. Thereafter, several diabetes care programs have studied the effectiveness of different types of diabetes management strategies. However, diabetes continues to rise and there remains a need to integrate specialist care into primary and community care settings for diabetes management, which is yet to be introduced into the healthcare systems/system-wide in most countries to address the growing burden of diabetes [6]. In some countries, integrated care models where specialist consultation is not covered by public health systems or health insurance companies can save out of pocket expenses of patients. In addition to potential improvement in clinical outcomes, it could also provide shared learning opportunities for primary healthcare professionals, reduce length of hospital stay and reduce duplication of service [8,9,10].

In this systematic review we focus on the active involvement of an endocrinologist (also known as diabetes specialist or diabetes specialist physician), where the endocrinologist works with the treating general practitioner (in some countries family physicians) and/or other healthcare professionals and helps guide patient diabetes management. The aim of this systematic review with meta-analysis was thus to provide an overview if integrated care for diabetes can improve clinical outcomes, effects on hospital admissions and cost-effectiveness specifically in integrated care interventions where endocrinologists have an active involvement within the primary care team. No reviews in the past have focused on the active role of endocrinologists in integrated care for diabetes.

In active involvement, the endocrinologist participates in the care of patients with diabetes along with the GPs and/or other healthcare professional and guides the team in a primary or intermediate care setting unlike in a specialist clinic where the patient is seen by an endocrinologist, without joint decision-making and with minimum communication between the primary care team and the endocrinologists [11,12]. Passive involvement is where the endocrinologist is not directly involved in the intervention such as providing regular training/workshops to GPs (provider education), expertise provided over an email or delivering advanced courses to the GPs [13,14].

## Methods

### Data Sources and Search

We followed the Preferred Reporting Items for Systematic Reviews and Meta-analysis (PRISMA) reporting guidelines for this systematic review and meta-analysis [15]. The protocol for our systematic review was registered on the International Prospective Register of Systematic Reviews, PROSPERO (registration number-CRD42019130968). MEDLINE, CINAHL, EMBASE, SCOPUS, Cochrane Library, and Google Scholar were searched for all eligible articles published until January 2021. We contacted experts in integrated diabetes care for grey literature (including any unpublished reports), research and public/government health departments in the following countries – Australia, the United States of America, Canada, the United Kingdom, and New Zealand. Peer-reviewed full text studies and research reports published in English language were included. We used a combination of synonyms and Medical Subject Headings (MeSH) search terms combined with Boolean operators (e.g., OR, AND, NOT), with the following keywords: diabetes mellitus, integrated health care systems, clinical outcome, and multidisciplinary team (Supplementary table 1 details search terms).

### Study selection

We included RCTs and pre-post intervention studies testing the effectiveness of integrated diabetes care interventions. Participants were non-pregnant adults (age ≥ 18 years) with type 1 or type 2 diabetes mellitus. Interventions had to have active endocrinologist involvement where the patients’ cases were discussed in a joint consultation with an endocrinologist along with a general practitioner and/or a third healthcare professional in primary care. Trial/intervention length had to be ≥6 months, allowing for 2 “HbA1c” cycles (HbA1c provides a measure of glycaemia over the prior 3 months). The outcomes of interest had to be change in glycaemia over time in intervention versus control groups in RCTs and between pre-post intervention in single group studies, as well as other key metrics such as systolic blood pressure (SBP), diastolic blood pressure (DBP), total cholesterol, weight and BMI, effect on hospital admissions and cost-effectiveness.

We excluded studies with interventions <6 months, not in English language, with published retractions or erratum where results were invalidated, studies reporting on outcomes in those <18 years of age, studies not including at least one clinical outcome and/or cost effectiveness analysis. Non-experimental studies were those (for example studies not including a pre-post introduction of a service measure), including passive endocrinologist involvement and studies only involving patient education/empowerment approaches. Studies not involving interventions within primary care contexts were excluded.

Two reviewers (RZ, JI) independently screened titles and abstracts, and resolved discrepancies through discussion with two other authors (DS, FM). Full-texts were screened and any discrepancies were resolved and agreed upon a final set of studies to be included. RZ contacted the experts in integrated diabetes care research and requested to share any grey literature (government or research reports, conference proceedings) on integrated diabetes care in that country (published or unpublished).

### Outcomes, Data extraction and Quality Assessment

Data were extracted by two authors (RZ, JI) for the following items: first author, year of publication, country of study, study type/design, type of diabetes, sample size, age, duration of study, care providers delivering interventions, interventions provided, outcome measures and results. The primary outcome was change in glycaemic control (HbA1c) from baseline to the last follow-up. For secondary outcomes, we extracted the change in systolic blood pressure (SBP), diastolic blood pressure (DBP), blood lipids, weight, BMI, cost-effectiveness and hypoglycaemia.

If studies did not report data of interest for the meta-analysis, corresponding authors were contacted where possible to obtain data. Data were analysed separately for RCTs and pre-post studies with and without control groups. The quality of selected studies was assessed independently by two authors (RZ, JI) using The National Institutes of Health study quality assessment tools for randomised controlled trials and pre-post studies and case-control studies [16]. The quality assessment for each study was answered as “Good”, “fair” and “poor”.

### Data Synthesis and Analysis

The study findings not included in the meta-analysis were summarized narratively. Estimates of cost outcomes for cost-effectiveness studies were not pooled as the outcomes were not comparable across studies. Where pooled analyses could be performed across randomised controlled trial or pre-post studies, data were included in meta-analyses. Where pre and post samples (n) differed, the post sample (n) was used for analysis as these were the only participants who would have given both pre and post measurements. Further, when there were less than four studies per outcome, no pooled analyses were carried out. Meta-analysis were performed using the *Metan* module in STATA version 15 software [17]. Mean differences and their standard deviations from each study were pooled using a random effects model using the procedure described by DerSimonian and Laird [18].

The pooled effect size was reported as a weighted mean difference (WMD) with its 95% CI, to enable easier interpretation. Heterogeneity was assessed using *I^2^* statistics, with values between 50–100% indicating substantial to considerable heterogeneity. The 27-item PRISMA checklist was used to guide the conduct and reporting of this systematic review [15].

### Publication Bias

The Doi plots method and the Luis Furuya-Kanamori (LFK) index were used to assess asymmetry of study effects in the plots [19] for meta-analyses including ≥5 studies.

## Results

### Search Results

**Figure 1:**
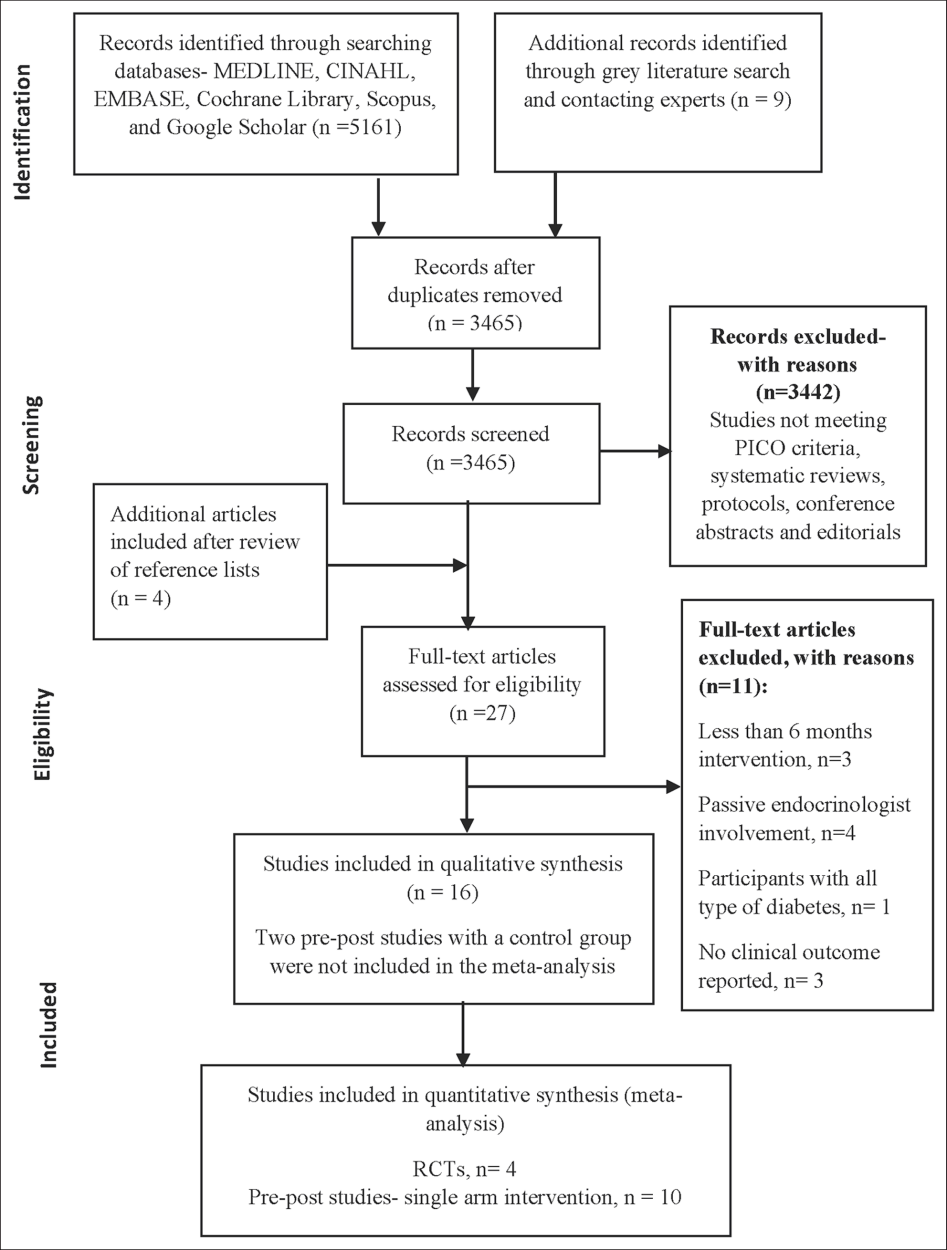
PRISMA flowchart diagram for studies selection (based on four-phase PRISMA flowchart diagram).

The study selection process was based on a four-phase PRISMA flow diagram and is reported in ***Figure 1*** commencing with 5,161 articles identified in the initial search leading to 16 studies meeting the inclusion criteria: 4 RCTs [11,12,20,21] and 12 pre-post studies [22,23,24,25,26,27,28,29,30,31,32,33].

### Study Characteristics

**Figure 2:**
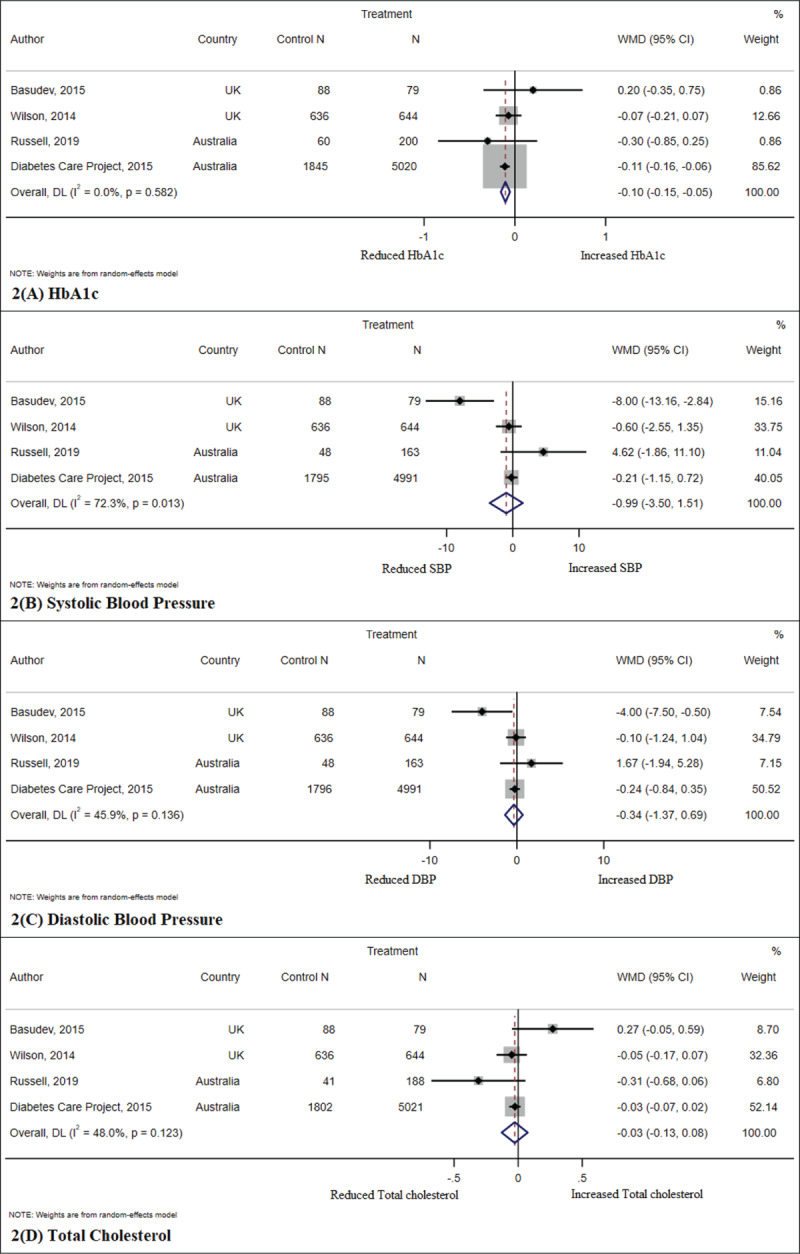
Forest plots for randomised controlled trials clinical outcomes. The results are expressed in WMD – weighted mean difference with 95% confidence intervals.

**Figure 3:**
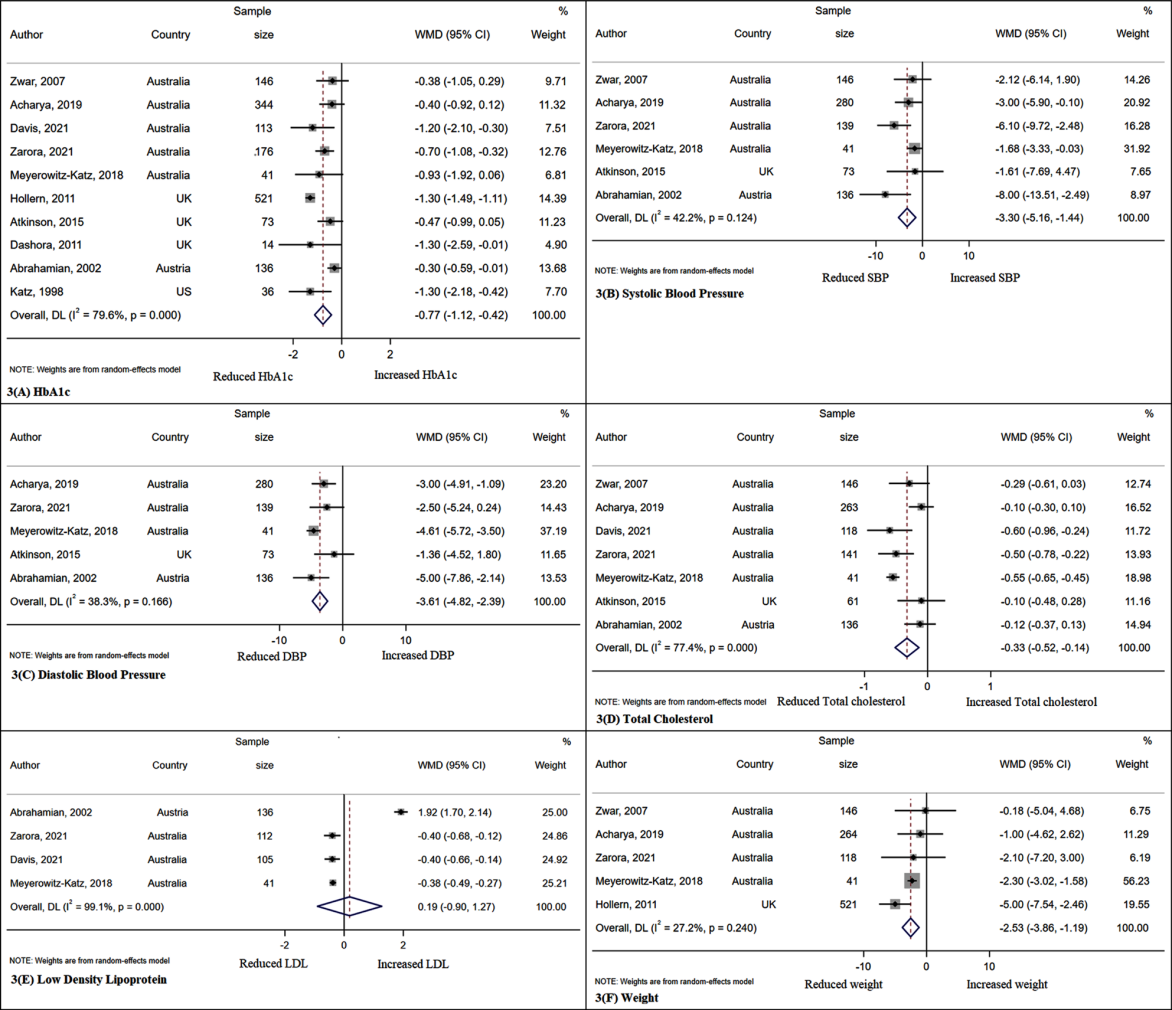
Forest plots for pre-post studies clinical outcomes. The results are expressed in WMD – weighted mean difference with 95% confidence intervals.

Characteristics of studies included are summarised in ***Table 1***. Nine studies were from Australia [12,20,22,23,25,28,30,32,33], five from the UK [11,21,26,27,29], one from the US [31] and one from Austria [24]. The duration of interventions lasted between 6–72 months. Twelve studies included people with type 2 diabetes [11,12,21,22,23,24,25,27,28,30,32,33] and four included people with both type 1 and type 2 diabetes [20,26,29,31]. The interventions varied between studies. All studies had an active general practitioner (primary care) and endocrinologist/diabetologist involvement (secondary care) and/or a third healthcare professional (dietitian, diabetes nurse educator, pharmacist, practice nurse, mental health worker, podiatrist, exercise physiologist). Standard diabetes care was provided by the general practice team in the control/usual group in the RCTs and pre-post studies with a control group. ***Figures 2*** and ***3*** include forest plots for the meta-analyses showing the effect of the intervention on clinical outcomes in RCTs and pre-post studies. Supplementary figure 1 shows Doi plots and the Luis Furuya-Kanamori (LFK) index to assess asymmetry for both primary and secondary outcomes.

**Table 1:** Characteristics of included studies in the systematic review (n = 16).


AUTHOR (YEAR), COUNTRY	STUDY POPULATION	STUDY TYPE	SAMPLE SIZE	AGE	DURATION OF STUDY	CARE PROVIDERS INVOLVED	INTERVENTION BY CARE PROVIDERS	OUTCOME MEASURES

Anthony W Russell (2019), Australia	Individuals with type 2 diabetes	A non-inferiority randomised controlled trial	Intervention group (n = 234) Control group (n = 71)	Age (years)- mean (SD) Intervention group 55.8 (11.3), Control group 55.4 (12.1)	33 months (2012–2015)	Two GPs with a special interest (GPwSIs), an endocrinologist and a diabetes nurse educator (DNE)	The single endocrinologist supervised and co-consulted with GPwSIs. The DNE was skilled in case co-ordination and worked independently between clinics. Duration of diabetes (years), mean (SD) Control group- 9.5 (7.8) Intervention group- 10.2 (8.1)	HbA1c, Blood Pressure, Cholesterol, BMI

N.Basudev (2015), United Kingdom	Individuals with type 2 diabetes	Prospective randomised controlled trial	Intervention group (n = 79) Control group (n = 88)	Age (years)- mean (SD) Intervention group 60.5(12.3), Control group 63(9)	12 months	Diabetes specialist nurses, a Diabetologist and a GP with special interest in diabetes	Integrated working between primary care and specialist diabetes teams were based on a ‘virtual clinic’ (professional-to-professional consultations- specialist to generalist- the patient is absent). Duration of diabetes (years), mean (SD) Control- 9.4(5.2) Intervention- 10.7(6.8)	HbA1c, Blood Pressure, Cholesterol, BMI, weight, eGFR

Andrew Wilson (2014), United Kingdom	Individuals with type 2 diabetes	Cluster-randomised trial	Intervention group (n = 644), Control group (n = 636)	Not stated	18 months	Consultant, General practitioner with Special Interest, Diabetes specialist nurse	Patients were reviewed and managed in the ICCD by a specialist nurse and a diabetologist with a goal of improving diabetes and cardiovascular risk factor and are then referred back to practice. Duration of diabetes was not reported.	HbA1c, Blood Pressure, Cholesterol

Diabetes Care Project (2015), Australia	Individuals with type 1 and type 2 diabetes	Cluster randomized controlled trial	Intervention group 1 (n = 2449), intervention group 2 (n = 2339) and Control group (n = 1845)	Not stated	18 months	General Practitioners, dietitians, pharmacists, practice nurses, endocrinologists, mental health workers, podiatrists, exercise physiologists	In Group 1 and Group 2 interventions, a collaboration between GPs and specialists undertook reviews of patient cases together. Following major changes were tested across the two groups: Integrated information platform, continuous quality improvement processes, flexible funding based on risk stratification, quality improvement support payments (QISP), funding for care facilitation. Duration of diabetes was equal to or more than 12 months’ duration.	HbA1c, Blood Pressure, Cholesterol, Serum Creatinine, GFR, ACR, weight, BMI.

Shamasunder Acharya (2019), Australia	Individuals with type 2 diabetes	Pre and Post study	n = 344	Age (years)- mean (SD) 63.2 ± 11.5	6 months	GP, Practice Nurse, Diabetes Educator and Endocrinologist.	Case-conference style consultations of 40-minute duration with 10 patients per day were conducted in the general practice with their own GP, Practice nurse, a visiting diabetes educator and an endocrinologist. Preparatory work included organising podiatry and eye review, up-to-date pathology. Diabetes duration (years) was- 9 (5 – 15)	HbA1c, Blood Pressure, Cholesterol, BMI, Weight, eGFR, Urine ACR

Gideon Meyerowitz-Katz (2018), Australia	Individuals with type 2 diabetes	Longitudinal pre-post single cohort design	n = 41	Age (years)- mean (SD) 56.46 (14.60)	3 years	Diabetologist, Registrar, Resident, and Nurse Educator.	Patient visits consisted of a joint patient consultation and multi-disciplinary case conference. The management plan was reviewed together and agreed between all participants and a report and treatment plan is generated. GPs were provided with a telephone support line for remote support for their decision-making. Duration of diabetes was not reported.	HbA1c, Blood Pressure, Cholesterol, Weight, eGFR,

Nicholas A Zwar (2007), Australia	Individuals with type 2 diabetes	Retrospective before and after study	n = 230	Age (years)- mean (SD) 61.2(11.4)	12 months	GP, Podiatrist, Diabetes Educator, Dietician, Endocrinologist, Ophthalmologist, Optometrist.	Multidisciplinary care for patients with diabetes was by at least two care providers other than the GP and at least one of these was diabetes related (e.g. Podiatrist, Diabetes Educator, Dietician, Endocrinologist, Ophthalmologist, Optometrist). Diabetes duration (years), mean (SD) was 6.7(6.1)	HbA1c, Blood Pressure, Cholesterol, Weight,

Heidemarie Abrahamian (2002), Austria	Individuals with type 2 diabetes	Prospective interventional study	n = 136	Mean (SD) age (years) at baseline (n = 154) – 69.2 (11.1). Patients completing the study (n = 136) Mean (SD) age (years) 69.1 (11.0)	12 months	Diabetes Specialist and four General Practitioners	Patients in whom the goals of treatment were not achieved or patients who developed acute complications were introduced by the GPs to the specialist via videoconferencing. The teleconsultations were not intended to exceed 15 min per patient. Diabetes duration (years), mean (SD) was 11.6 (10.7).	HbA1c, Blood Pressure, Cholesterol

Rosarie Atkinson (2015), United Kingdom	Type 1 –7 (8%) Type 2- 106 (92%)	A prospective clinical audit	n = 73	Mean (SD) age (years) 59.15(14.57) Age groups 0–29 years- 2 (2%) 30–49 years- 34 (30%) 50–69 years- 44 (39%) 70 years and above- 33 (29%)	6 months	Diabetes Specialist nurses, a Diabetologist and a General Practitioner with special interest in diabetes	The components of the diabetes virtual clinic (DVC) were: systematic case identification; a virtual clinic in which cases (n = 15 to 20) were jointly discussed by the GP and DVC teams- to determine clinical and therapeutic needs, self-management needs and the most appropriate care provider; formulation of a management plan; a face-to-face appointment with the most appropriate member of the clinical team to develop an agreed care plan. Duration of diabetes was not reported.	HbA1c, Blood Pressure, Cholesterol, BMI, eGFR, Albumin creatinine ratio

Timothy M E Davis (2021), Australia	Individuals with type 2 diabetes	Single-arm intervention study	n = 113	Mean (SD) age (years) 59.3±12.2	27 months	An upskilled GP, diabetes nurse educator and endocrinologist.	Each DCCC participant was assessed by an upskilled GP and a management plan was developed in consultation with an endocrinologist who also reviewed the participant if appropriate. The management plan was then discussed with the patient and the DCCC DNE, and communicated to the participant’s usual GP. Diabetes duration (years) was-10 [4,5,6,7,8,9,10,11,12,13,14,15,16]	HbA1c, Blood Pressure, Cholesterol, BMI

Helen Hollern (2011), United Kingdom	Individuals with Type 1 and Type 2 diabetes	Pre and Post study	n = 521	Not stated	6 months	Diabetes specialist nurses, specialist podiatrist, specialist dietitian, care technicians, consultant diabetologist, PA/admin support.	GP practice staff and specialist team met in a virtual clinic to discuss individuals and made suggestions to possible changes in treatment or lifestyle. One or more members of the specialist team attended each virtual clinic. Duration of diabetes was not reported.	HbA1c, Weight

Umesh Dashora (2011), United Kingdom	Individuals with type 2 diabetes	Pre and Post study	n = 15	Mean (SD) age (years) 49.5 (22.0)	6 months	GP, the consultant and the diabetes specialist nurse.	Individuals were jointly seen by the GP, the consultant and the DSN together. Participants were able to discuss their diabetes control with the doctors and have their treatment adjusted. They were referred to other services as required. Duration of diabetes was not reported.	HbA1c

Reetu Zarora (2021), Australia	Individuals with type 2 diabetes	Pre and Post study	n = 178	Mean (SD) age (years) 65±11	2.5 years	Endocrinologist, Dietitian, Credentialed diabetes educator, and Podiatrist	A monthly specialist clinic led by an endocrinologist supported by a dietitian and credentialed diabetes educator in the local community health centre involved face-to-face clinical review of patients with Type 2 diabetes requiring specialist advice and weekly dietetic, diabetes educator, group education and foot-screening clinic. Diabetes duration (years) was- 19 (11.0–24.0)	HbA1c, Blood Pressure, Cholesterol, BMI, Weight, eGFR,

Gillian Katz (1998), United States	Individuals with Type 1 and Type 2 diabetes	Pre and Post study	n = 36	Mean (±SD) 54.6 ± 11.2 years.	12 months	Diabetologist, a bicultural certified diabetes nurse-educator, and a nutritionist,	Referral were made by the primary care physicians. A complete assessment of diabetes knowledge and self-care skills was undertaken by the diabetes nurse-educator and the dietitian provided nutritional counselling. Each patient had a consultation with the diabetologist; a complete medical history was elicited, and a physical examination was performed. Diabetes duration (years), mean (SD) was 11.2(9.9).	HbA1c

Claire Jackson (2010), Australia	Individuals with type 2 diabetes	Pre and Post study with control arm	Intervention group (n = 99) and Usual care group (n = 67)	Not stated	12 months	Endocrinologist, advanced skilled GPs known as ‘clinical fellows’, a credentialed diabetes educator and a podiatrist.	All patients were first assessed by a clinical fellow who examines the patient, interprets the retinal photographs and pathology results, and drafts a management plan and patient priorities. The plan is discussed with the attending endocrinologist, who then co-consults with the patient and clinical fellow together to finalise the approach. Duration of diabetes was not reported.	HbA1c

Anthony W. Russell (2013), Australia	Individuals with type 2 diabetes	Prospective open controlled trial	Intervention group (n = 127), Usual care group (n = 121)	Age (years)- mean (SD) Intervention group- 59.4(13.4) years, Usual group- 62.9(11.6)	12 months	GP Clinical Fellows, a GP training registrar, an endocrinologist, diabetes educator, dietician, psychologist and podiatrist.	At the initial visit, patients underwent a 45-min comprehensive screening and then attended the ICDMS multidisciplinary clinic. The GP Clinical Fellow briefly consulted the endocrinologist to review the management plan and then both co-consulted with the patient to finalise the management approach. Duration of diabetes (years), mean (SD)- Control group 13.7 (10.2), Intervention group 12.8 (9.7)	HbA1c, Blood Pressure, Cholesterol, BMI, eGFR, Serum creatinine


**M**- Mean, **ICCD**- Intermediate care clinics for diabetes, **GP**- General Practitioner, **GPwSIs**- General Practitioners with a special interest, **ACR**- Albumin Creatinine Ratio, **DCCC**- Diabetes Complex Collaborative Care project, **DSN**- Diabetes Specialist Nurse, **ICDMS**- Inala Chronic Disease Management Service, **DNE**- Diabetes Nurse Educator.

### Meta-analysis

Findings suggest that integrated care models of diabetes specialists working with primary care health professionals had a positive impact on HbA1c in both RCTs and pre-post studies and on systolic blood pressure, diastolic blood pressure, total cholesterol and weight in pre-post studies. HbA1c, SBP, DBP and total cholesterol were lower in both post intervention RCTs groups and pre-post studies (***Tables 2*** and ***3***). There was a reduction in weight and an increase in the LDL in the pre-post studies.

**Table 2:** Pooled analysis of studies across RCTs in the intervention group.


CLINICAL VARIABLE	NUMBER OF STUDIES	MEAN DIFFERENCE	95% CONFIDENCE INTERVAL	HETEROGENEITY (*I^2^*)

HbA1c (%)	4	–0.10	–0.15 to –0.05	0%

SBP (mmHg)	4	–0.99	–3.50 to 1.15	72.3%

DBP (mmHg)	4	–0.34	–1.37 to 0.69	45.9%

Total cholesterol (mmol/L)	4	–0.03	–0.13 to 0.08	48%


**Table 3:** Pooled analysis of pre-post studies.


CLINICAL VARIABLE	NUMBER OF STUDIES	MEAN DIFFERENCE	95% CONFIDENCE INTERVAL	HETEROGENEITY (*I^2^*)

HbA1c (%)	10	–0.77	–1.12 to –0.42	79.6%

SBP (mmHg)	6	–3.30	–5.16 to –1.44	42.2%

DBP (mmHg)	5	–3.61	–4.82 to –2.39	38.3%

Total cholesterol (mmol/L)	7	–0.33	–0.52 to –0.14	77.4%

Weight (kg)	5	–2.53	–3.86 to –1.19	27.2%

Low-density lipoprotein (mmol/L)	4	0.19	–0.90 to 1.27	99.1%


***Figures 2*** and ***3*** shows the forest plot for clinical outcomes for RCTs and pre-post studies and the studies included in the analysis of each clinical outcome variable.

The Doi plot with LFK index (supplementary figure 1) showed minor to major asymmetry for all outcomes except for diastolic and systolic blood pressure. Because, for all outcomes, the number of included studies was small, there were no further analyses to explore the cause of the asymmetry. In the meta-analyses of RCTs, there was low heterogeneity in all outcomes expect for systolic blood pressure with an I^2^ of 72.2%. In the meta-analyses of pre-post studies, there was also low heterogeneity in all outcomes expect for LDL-Cholesterol, total cholesterol and HbA1c. Again, it was not possible to explore the causes of the heterogeneity to the low number of included studies.

### Narrative findings

Among two pre-post studies with a control group, HbA1c decreased significantly in one study [32] after 12 months by 1.4% (15.3 mmol/mol) (p-value = 0.0001). Among pre-post studies with no control group, one reported significant reductions in mean BMI (0.8 kg/m^2^) (p-value <0.001) [23] and two studies reported statistically significant reductions in triglycerides over 12 months (–0.2 mmol/L) (p-value = 0.005) and 36 months (–0.28 mmol/L) (p-value = 0.029) [28,30].

#### Hypoglycaemia

In a pre-post study with no control group 28% patients experienced hypoglycaemia at baseline and only 4% after one year of intervention (p-value < 0.001) [31].

#### Economic Outcomes

A UK pre-post study reported a total cost saving of 125,925 GBP after one year from changes in medication and in admission cost [29]. In an RCT in Australia, the intervention groups’ model of care was not cost-effective compared to the control group. The cost in Group 1 and Group 2 increased by 718 AUD and 203 AUD (p = 0.275 and p = 0.758) per person per annum respectively [20]. Similarly, the intervention group in another RCT was marginally more expensive than the control group in another study from the UK [21]. Estimates of cost outcomes for cost-effectiveness studies were not pooled as the outcomes were not comparable across trials/studies.

#### Hospitalisation Outcomes

The number of hospitalisations in one pre-post study reduced from 12 before the intervention to 7 in the year of the project for acute diabetes complications and duration of hospitalisation reduced from 110 to 68 days per year for acute complications treatment [24].

### Quality assessment

Of the 4 RCTs in the systematic review, two were classified as good quality [11,12] and 2 were classified as fair quality [20,21] overall. Of the 12 pre-post studies included, 5 were reported as good quality [24,25,28,30,33] and 7 were reported as fair [22,23,26,27,29,31,32] overall. In two RCTs, participants and providers were not blinded to treatment group assignment [20,21], which could bias the results due to poor allocation concealment. In 3 RCTs people assessing the outcomes were not blinded to the participants’ group assignments [12,20,21]. Only one pre-post study reported outcome measures multiple times after the intervention [30] and none of the pre-post studies reported if people assessing the outcomes were blinded to the participants’ exposures/interventions. In 4 pre-post studies the sample size was not sufficiently large to provide confidence in the findings [26,27,30,31]. The loss to follow-up in six pre-post studies after baseline was 20% or less [22,23,25,26,29,31]. In both pre-post studies with a control group, the assessors of exposure/risk were not blinded to the case or control status of participants [32,33]. High risk of performance bias was observed in the pre-post studies with and without control group, as the participants and care providers were not blinded to the intervention.

## Discussion

This systematic review and meta-analysis provides evidence that integrated diabetes care with active endocrinologist roles likely improved clinical outcomes in a range of settings. Meta-analysis of both RCTs and pre-post studies showed reductions in HbA1c and pre-post studies also showed reductions in systolic blood pressure, diastolic blood pressure, total cholesterol and weight. However, heterogeneity of the included studies suggest that findings should be treated with caution and that further studies, including RCTs, are required. Interventions in the included studies involved integrated working between primary care and specialist diabetes teams. Multidisciplinary care for patients with diabetes included at least two care providers (an endocrinologist and a GP) and in most studies a diabetes related third healthcare professional (e.g., Diabetes Nurse, Podiatrist, Diabetes Nurse Educator, Diabetes Educator, Dietician, Ophthalmologist, Optometrist). Patients were reviewed and managed with a goal of improving diabetes with ongoing care provided by primary care. One trial included an integrated information platform, continuous quality improvement processes, flexible funding based on risk stratification, quality improvement support payments (QISP) and funding for care facilitation [20]. In some studies, patients were included in a joint consultation with the multi-disciplinary team. The management plan was reviewed together and agreed between all participants and a report and treatment plan was generated [25,27,28,30,31,33]. GPs were provided with a videoconference consultation support line for remote support for their decision-making in one study [24]. Some were virtual clinics, where patients were introduced by the GPs to the specialist and cases were jointly discussed by the GP and the specialist team, to determine clinical and therapeutic needs, self-management needs, formulation of a management plan and a face-to-face appointment with the most appropriate member of the clinical team to develop an agreed care plan [11,29].

Most of the included studies in this systematic review focused on type 2 diabetes and only 4 included both type 1 and type 2 diabetes. Newer technologies continue to emerge for the management of type 1 diabetes, however, the biggest challenge remains to manage the hypoglycaemia and hyperglycaemia episodes in people with type 1 diabetes. Type 1 diabetes management is largely a specialist task and it remains important for primary and secondary care to work together for the holistic management of these patients. It is not necessary that GPs would have all knowledge and skills to treat diabetes and its complications and thus multidisciplinary input is an opportunity for shared learning and decision-making.

Previously conducted systematic reviews studied a range of interventions and the involvement of an endocrinologist in patient diabetes management was either passive, limited or absent. One systematic review assessed the effects of healthcare professional interventions on the management of diabetes in patients where the effect on patient outcomes was less clear as these were rarely assessed, [34]. Another systematic review focused on different types of integrated care interventions and their outcomes, where most interventions included all components of the Chronic Care Model [35] with limited or no endocrinologist involvement. Patient outcome measures were reported by a small number of included articles and reported as positive effects on clinical outcome measures (improvement in glycaemic control, blood pressure, cholesterol, and BMI) and improvements in process measure [35]. One meta-analysis studied the effectiveness of multicomponent integrated care including peer-support and e-health on clinical outcomes in patients with type 2 diabetes and reported improvements in clinical outcomes (reduced HbA1c by 0.28%, reduced SBP by 2.3 mmHg and DBP by –1.1 mmHg), however the involvement of endocrinologists in a multidisciplinary team in patient care was either passive, limited or absent [36]. Other meta-analysis evaluated clinical outcomes of patients with uncontrolled diabetes managed by a multidisciplinary team of care providers (care provided by two different healthcare professionals) where pooled studies reported improved clinical outcomes (reduced HbA1c by –0.55%, reduced SBP –4.89 mmHg and DBP by 2.3 mmHg) on patient’s blood glucose and systolic blood pressure [37]. The two care providers in the included studies who managed patients’ diabetes were mostly a pharmacist or a nurse/diabetes nurse and did not include an endocrinologist.

### Effect on diabetes related hospitalisation

Findings from a study in Australia suggest that hospitalisation related to diabetes complications can be prevented/avoided and significant savings can be made when patients receive care from a multidisciplinary specialist team. It reported an estimated national cost savings of AUD 132.5 million per year and an average number per patient reduction (0.19) in potentially preventable hospitalisation rates for diabetes related complication admissions in the intervention group (n = 182) over a 24 month period [38]. In another study, patients in the intervention group were nearly half as likely to be hospitalised for a potentially preventable diabetes related principal diagnosis compared to the usual group after study commencement. Patients in the usual care group were hospitalised for significantly longer than intervention groups when the principal diagnosis was a diabetes-related complication (potentially preventable hospitalisation) and both had similar length of stay when hospitalised (median difference –2 days, 95%CI –6.5, 2.3; p-value = 0.33) [8]. A structured model of integrated care (integrating primary and secondary care) adhering to the guidelines can be less expensive and reduce hospitalisation than usual care for type 2 diabetes.

### Cost-effectiveness of integrated diabetes care programs

Integrated care programmes have the potential to be cost-effective. Cost savings/economic impact were reported in an integrated care study (a comprehensive health care management program where a multidisciplinary team worked with physicians and patients to improve clinical outcomes and short-term savings) in the United States, where the total costs decreased by 44 USD per patient with diabetes per month (10.9%) and in another study, reduction in hospitalisation where inpatient hospital costs fell by 47 USD per patient with diabetes per month [9].

### Role of primary care team in diabetes care

There is diversity in the standards of clinical practice/healthcare systems in different countries. General practitioners may have ultimate responsibility for overall patient management, and endocrinologists’ involvement is important to manage more complex diabetes and to help achieve glycaemic targets for patients where additional assistance is required. GP’s can better manage people with type 1 and complex type 2 diabetes by involving diabetes specialist professionals within the practice or referring to a diabetes specialist physician outside the practice. In the Pittsburgh Epidemiology of Diabetes Complications Study, a lower HbA1c level 9.7 vs 10.3% (82.5 mmol/mol vs 89.1 mmol/mol) was associated with specialist care versus generalist care in a cohort of patients with type 1 diabetes [39]. Conversely, general practitioners can review clinical arrangement for patients unable to attend specialist clinics [40].

### Healthcare professional up-skilling

Some studies included in this systematic review focused on both managing patients and healthcare professional upskilling. These interventions allowed transfer of knowledge from specialist to the GP and the primary care team, providing learning opportunities with real case-based discussion and with knowledge specific to the needs of patients and allowing the specialist to identify and fill gaps in knowledge as appropriate [23,27]. One study developed a series of interactive educational sessions covering relevant and contemporary topics in diabetes. Wider benefits included partnership and trust building between specialist and primary care, which allowed timely referral to specialist services when required. Practice nurses and GPs reported increased competency and confidence in treatment escalation [25]. Another study focused on improving practice capacity to manage diabetes care by up-skilling primary care providers (GPs, practice nurses) [30].

### Funding for integrated diabetes care programs

The budget allocation determines the duration of the care provided, out of pocket fee for the patient and involvement of other specialist professionals [23,41,42]. Findings from previous studies show that a multidisciplinary team involving a GP, endocrinologist, nurse, allied health professional (diabetes educator, dietitian, podiatrist) and psychologist, can better manage patients with diabetes compared to usual care [43,44,45]. Several GP and endocrinologist led and other allied health intervention studies involving an endocrinologist have been published globally showing improvements in clinical and cost-effectiveness outcomes and reductions in hypoglycaemic episodes including in indigenous patients with diabetes in rural settings in Australia [46,47,48]. Some studies were government or hospital trust-funded for workforce recruitment including diabetes specialist, diabetes educator, project officer, podiatrist, dietitian, administrative officer and data extraction and analysis costs [23,25,29,31].

### Strengths and Limitations

This is the first study to evaluate the impact of multidisciplinary integrated diabetes care where endocrinologists had an active involvement in patients’ diabetes care and guided the diabetes management plan. This systematic review examined studies conducted in different geographical areas such as America, Europe and Australia. This study followed the PRISMA reporting guidelines including a 27-item checklist and a four-phase flow diagram which includes essential items for transparent reporting of a systematic review. This improves the clarity as to how the review was conducted. The study protocol was registered with PROSPERO to provide transparency, reduce potential bias and the unintended duplication of reviews. Two researchers independently conducted the search to eliminate bias, avoid systematic errors in methodology, avoid missing eligible studies which added to the quality of review. Previous systematic reviews have included only RCTs in the meta-analysis, however, this systematic review includes both experimental- RCTs (n = 4) and non-experimental- single group pre-post studies (n = 10) in the meta-analysis. Complex pre-post interventions where data are collected at different time points also increases the methodological rigour [49].

There were several limitations in this systematic review: only English language articles were included thus some relevant articles may have been excluded. Limited studies met inclusion criteria, as there was a lack of uniformity in the definition of integrated care. Due to the limited number of studies on the impact of interventions on cost-effectiveness, generalizability of cost-effectiveness findings to these countries is limited. There was considerable heterogeneity between the studies which could be due to the interventions being delivered in different healthcare settings, variation in treatment effects, duration of treatment, number of intervention components and degree of complexity of the diabetes.

## Conclusion

This systematic review and meta-analysis shows that people with diabetes can be managed/treated better within an integrated primary-secondary care approach with significant improvements in clinical and economic outcomes compared to usual care where the two sectors operate in a less coordinated manner. Such approaches can be helpful in settings with limited funding, where the GP works with specialists to treat people with type 1 and type 2 diabetes. Integrated diabetes care approaches varied, with opportunities of further studies including more diverse methods including “tools” to improve communication (e.g. digital approaches). There is room for more robust evaluation methods particularly health economic assessments. This paper has focused on integrated care that has been built upon the wider principles of multidisciplinary healthcare professional teams, patient diabetes self-management, and communication being key between the multidisciplinary team.

## Data Accessibility statement

The dataset generated during the study and analysed are available on request from the corresponding author.

## Additional File

The additional file for this article can be found as follows:

10.5334/ijic.6025.s1 Supplemental Tables and Figure. Tables 1 to 3 and Figure 1.
