# Supplementary material for: Effectiveness of Integrated Diabetes Care Interventions Involving Diabetes Specialists Working in Primary and Community Care Settings: A Systematic Review and Meta-Analysis
Source: Int J Integr Care. 2022 May 12;22(2):11. doi: 10.5334/ijic.6025 (PMC9104489; doi:10.5334/ijic.6025)
Supplement: Supplemental Tables and Figure. — Tables 1 to 3 and Figure 1. [file ijic-22-2-6025-s1.pdf]

Supplementary table 1. Search terms and combination MeSH terms used for each database.

**Search Terms**	INTERVENTION<br><br>Integrated Health Care Systems, Integrated Delivery System, Integrated care, Multidisciplinary care, Multidisciplinary team, Telehealth OR Self-management OR Peer support<br><br>POPULATION<br><br>Diabetes, Type 1 diabetes, Type 2 diabetes, Diabetes mellitus<br><br>OUTCOME<br><br>Clinical outcome, Patient outcome, Quality improvement, Cost effectiveness, Cost-Benefit Analysis, Economic evaluation, Costs and Benefits
**Combinations**	Integrated Health Care Systems OR Integrated Delivery System OR Integrated care OR Multidisciplinary care OR Multidisciplinary team OR Telehealth OR Self-management OR Peer support AND Type 1 diabetes OR Type 2 diabetes OR Diabetes OR Diabetes Mellitus AND Clinical outcome OR Patient outcome OR Quality improvement OR Cost effectiveness OR Cost Benefit Analysis OR Economic evaluation OR Costs and Benefits

Supplementary table 2: Search Strategy for e.g. for CINAHL Plus

#	Query	Limiters/Expanders	Last Run Via	Results
S1	AB Integrated Health Care Systems OR AB Integrated Delivery System OR AB Integrated care OR AB Multidisciplinary care OR AB Multidisciplinary team OR AB Telehealth OR Self-management OR Peer support	Search modes - Boolean/Phrase	Interface - EBSCOhost Research Databases<br>Search Screen -Advanced<br>Search Database - CINAHL Plus	34,829
S2	AB Diabetes OR AB Type 1 diabetes OR AB Type 2 diabetes OR AB Diabetes mellitus	Search modes - Boolean/Phrase	Interface - EBSCOhost Research Databases<br>Search Screen -Advanced<br>Search Database - CINAHL Plus	110,314
S3	AB Clinical outcome OR AB Patient outcome OR AB Quality improvement OR AB Cost effectiveness OR AB Cost-Benefit Analysis OR AB Economic evaluation OR AB Costs and Benefits	Search modes - Boolean/Phrase	Interface - EBSCOhost Research Databases<br>Search Screen -Advanced<br>Search Database - CINAHL Plus	192,964
S4	S1 AND S2 AND S3	Search modes - Boolean/Phrase	Interface - EBSCOhost Research Databases<br>Search Screen -Advanced<br>Search Database - CINAHL Plus	790
S5	S1 AND S2 AND S3	Narrow by Language: - **English**<br>Search modes - Boolean/Phrase	Interface – EBSCOhost Research Databases<br>Search Screen – Advanced<br>Search Database - CINAHL Plus	787

Supplementary table 3: Reason for exclusion of studies at final screening

Author (Year), Country	Title	Journal	Reason for exclusion
Alan L Graber (2012), The United States	Improving glycemic control in adults with diabetes mellitus: shared responsibility in primary care practices.	Southern Medical Journal	Study duration less than 3 months
David Clarke (2002), New Zealand	Integrated disease management pilot for diabetes	Journal of Healthcare Information Management: JHIM	Study duration less than 3 months
David Simmons (2003), Australia	Impact of an integrated approach to diabetes care at the Rumbalara Aboriginal Health Service	Internal Medicine Journal	Study duration 3-24 months
Irl B. Hirsch (2002), The United States	A multifaceted intervention in support of diabetes treatment guidelines: a controlled trial	Diabetes Research and Clinical Practice	There was passive endocrinologist involvement in the care- regular teaching was provided to the family practice providers.
Gretchen A. Piatt (2010), The United States	3-Year Follow-up of Clinical and Behavioral Improvements Following a Multifaceted Diabetes Care Intervention	The Diabetes Educator	There was passive endocrinologist involvement in the care – patient and provider education.
Robert Stroebel (2005), The United States	Adapting the Chronic Care Model to Treat Chronic Illness at a Free Medical Clinic	Journal of Health Care for the Poor and Underserved	There was passive endocrinologist involvement in the care- speciality expertise was available by e-mail with the endocrinologist.
Larry A. Distiller (2009), South Africa	Striving for the impossible dream: a community-based multi-practice collaborative model of diabetes	Diabetic Medicine	There was passive endocrinologist involvement

	management		in the care as the endocrinologist delivered a comprehensive 3-Day Advanced Course.
Ulrike Rothe (2008), Germany	Evaluation of a Diabetes Management System Based on Practice Guidelines, Integrated Care, and Continuous Quality Management in a Federal State of Germany	Diabetes Care	Participants were patients with all types of diabetes.
Jianzhen Zhang (2015), Australia	Impact of an integrated model of care on potentially preventable hospitalizations for people with Type 2 diabetes mellitus	Diabetic Medicine	No clinical outcome reported
Samantha A. Hollingworth (2017), Australia	Impact of a general practitioner-led integrated model of care on the cost of potentially preventable diabetes-related hospitalisations	Primary Care Diabetes	No clinical outcome reported
F A McAlister (2007), Canada	The effect of specialist care within the first year on subsequent outcomes in 24,232 adults with new-onset diabetes mellitus: population-based cohort study	Specialist care in diabetes	No clinical outcome reported

Supplementary figure 1: *Doi* plot method and Luis Furuya-Kanamori (*LFK*) index used to detect and quantify asymmetry of study effects in the plots.

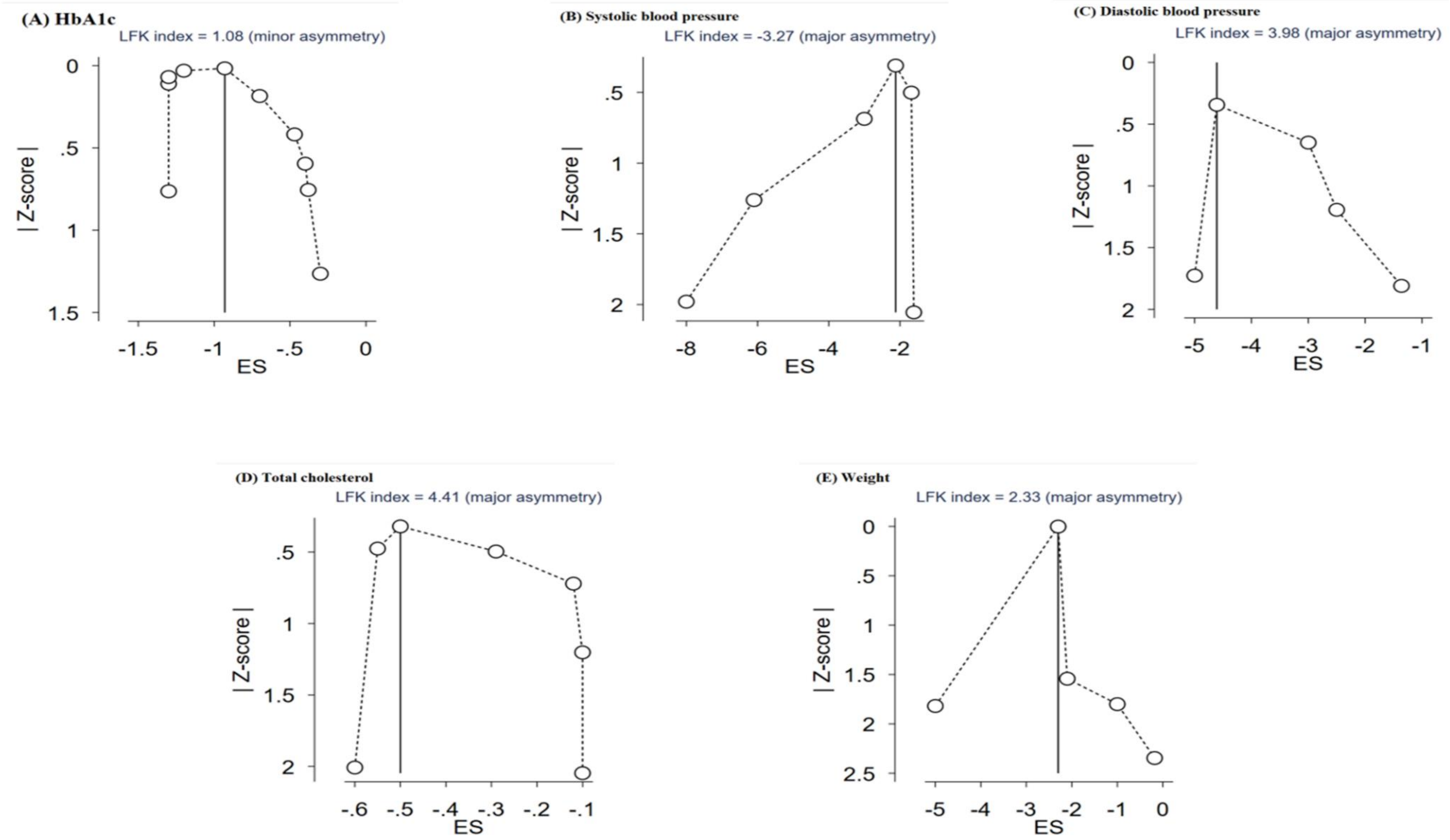
(A) HbA1c
LFK index = 1.08 (minor asymmetry)
| Z-score |
ES
(B) Systolic blood pressure
LFK index = -3.27 (major asymmetry)
| Z-score |
ES
(C) Diastolic blood pressure
LFK index = 3.98 (major asymmetry)
| Z-score |
ES
(D) Total cholesterol
LFK index = 4.41 (major asymmetry)
| Z-score |
ES
(E) Weight
LFK index = 2.33 (major asymmetry)
| Z-score |
ES
